# The Daughterless N-terminus directly mediates synergistic interactions with Notch transcription complexes via the SPS+A DNA transcription code

**DOI:** 10.1186/1756-0500-2-65

**Published:** 2009-04-28

**Authors:** John W Cave, Li Xia, Michael A Caudy

**Affiliations:** 1 Department of Neuroscience, Weill Cornell Medical College, New York, NY 10021, USA; 2 Burke Medical Research Institute, White Plains, NY, 10605, USA; 3 Current address: Gnomics Web Services, New York, NY, 10471, USA

## Abstract

**Background:**

Cell-specific expression of a subset of *Enhancer of split (E(spl)-C) *genes in proneural clusters is mediated by synergistic interactions between bHLH A (basic Helix-Loop-Helix Activator) and *Notch*-signalling transcription complex (NTC) proteins. For a some of these *E(spl)-C *genes, such as *m8*, these synergistic interactions are programmed by an "SPS+A" transcription code in the *cis*-regulatory regions. However, the molecular mechanisms underlying this synergistic interaction between NTCs and proneural bHLH A proteins are not fully understood.

**Findings:**

Using cell transcription assays, we show that the N-terminal region of the Daughterless (Da) bHLH A protein is critical for synergistic interactions with NTCs that activate the *E(spl)-C m8 *promoter. These assays also show that this interaction is dependent on the specific inverted repeat architecture of Suppressor of Hairless (Su(H)) binding sites in the SPS+A transcription code. Using protein-protein interaction assays, we show that two distinct regions within the Da N-terminus make a direct physical interaction with the NTC protein Su(H). Deletion of these interaction domains in Da creates a dominant negative protein that eliminates NTC-bHLH A transcriptional synergy on the *m8 *promoter. In addition, over-expression of this dominant negative Da protein disrupts Notch-mediated lateral inhibition during mechanosensory bristle neurogenesis *in vivo*.

**Conclusion:**

These findings indicate that direct physical interactions between Da-N and Su(H) are critical for the transcriptional synergy between NTC and bHLH A proteins on the *m8 *promoter. Our results also indicate that the orientation of the Su(H) binding sites in the SPS+A transcription code are critical for programming the interaction between Da-N and Su(H) proteins. Together, these findings provide insight into the molecular mechanisms by which the NTC synergistically interacts with bHLH A proteins to mediate *Notch *target gene expression in proneural clusters.

## Background

In *Drosophila*, neurogenesis is initiated by the expression of proneural bHLH A (basic Helix-Loop-Helix Activator) genes in "proneural clusters" of adjacent cells. Proneural bHLH A proteins, such as Achaete (Ac), heterodimerize with the ubiquitously expressed Daughterless bHLH A protein (Da) and activate target genes. Within proneural clusters, typically only one or a few cells become a neural precursor cell (NPC) and proneural bHLH A gene expression is upregulated within the NPC. By contrast, *Notch *signalling-mediated lateral inhibition represses bHLH A gene expression in the non-NPCs, where the Notch receptor is activated. This repression of bHLH A gene expression is mediated by several *Enhancer of split Complex (E(spl)-C) *proteins that are specifically upregulated in the non-NPCs.

The cell-specific expression of *E(spl)-C *genes in proneural clusters is mediated by a strong synergistic interaction between bHLH A and *Notch*-signalling transcription complex (NTC) proteins assembled on the *cis*-regulatory regions of the *E(spl)-C *genes [1,2]. For a specific subset of *E(spl)-C *genes, including *m8*, these synergistic interactions are programmed by an "SPS+A" transcription code. This transcription code contains proneural bHLH A protein binding sites ("A" sites), and an "SPS" DNA element (where "SPS" is a Su(H) Paired Site, an inverted repeat architecture of Suppressor of Hairless (Su(H)) binding sites [3,4]). Previous studies have shown that the inverted repeat orientation architecture of the SPS element is essential for the "NTC-bHLH A" synergy programmed by the SPS+A transcription code [1]. Genetic yeast two-hybrid assays in these previous studies suggested that the Da bHLH A and Su(H) proteins interact as part of NTC-bHLH A transcriptional synergy. In this study, we show that: 1) there is a direct physical interaction between the Da N-terminal domain and Su(H); 2) the Da N-terminus is sufficient for synergistic activation of *m8 *in cell culture transcription assays, but requires the proper SPS orientation architecture; and 3) the Da-N domain is essential for NTC-bHLH A transcriptional synergy which is required for proper Notch-mediated lateral inhibition *in vivo*.

## Methods

Details of the S2 cell culture transfection protocol have been described elsewhere [1]. All protein expression plasmids for S2 cell culture were constructed using pAc 5.1/V5-HisA plasmids (Invitrogen). The Da-N, Da-N1, Da-N2 and Da-bHLH coding sequences were generated by PCR amplification. All other expression plasmids used for S2 cell culture have been previously described [1]. All transcription reporters for S2 cell culture were constructed with pGL2-basic *luciferase *plasmids (Promega). Details about the construction of the native *m8-*WT, *m8*-RF and the artificial 4A and SPS-4A promoters have been given elsewhere [1]. Statistical analysis of luciferase assay data was performed using either T-test comparisons or ANOVA with appropriate post-hoc tests. Differences with probabilities of p < 0.01 were considered significant.

For the protein pull-down assays, 6xHis-tagged proteins were expressed using the pET-28A vector (Novagen) and 6xMyc-tagged proteins were expressed using a modified pET-21A (Novagen) vector that added 6 tandem repeats of the Myc epitope to the expressed proteins. Proteins were expressed in either pLysS or pACYC strains of BL21(DE) bacteria for 6 hours after induction with IPTG. Protein expression was confirmed with Western blot analysis using either α-6xHis (Novagen) or α-myc (Sigma) antibodies. Following IPTG-induction expression, cells were spun down, resuspended in 50 mM sodium phosphate (pH 8.0) containing 150 mM NaCl and 20 mM imidazole and then disrupted by sonication. Cellular debris following sonication was removed by centrifugation and 6xHis-tagged proteins were bound to equilibrated Ni-resin spin columns (Qiagen). Lysates containing 6xMyc-tagged proteins were then passed through the Ni-resin spin columns in which the 6xHis-tagged protein were bound. Columns were then treated with 50 mM Tris (pH 8.0) containing 10 mM EDTA to remove the 6xHis-tagged protein and any 6xMyc-tagged proteins with which they bound. Eluted proteins were analyzed by Western blot analysis using either α-6xHis or α-myc antibodies.

Yeast two-hybrid assays used the MatchMaker Yeast Two-hybrid system (Clontech). All "bait" and "prey" fusion protein constructs used the pGBKT7 and pGADT7 plasmids, respectively, provided by the MatchMaker system. Inserts containing the reading frames for the various Da and Ac proteins were shuttled from the pAc5.1 expression plasmids used for transcription assays in S2 cell culture. Interactions between bait and prey proteins were measured using β-galactosidase activity in transformed AH109 *S.cerevisae *cells provided by the MatchMaker system. Interaction assay values are reported relative to the activity of the bait construct with an empty pGADT7 vector. Statistical analysis of yeast two-hybrid assay data was performed using T-test comparisons, and differences with probabilities of p < 0.05 were considered significant.

Transgenic fly lines containing the various *UAS-Da *responder transposable elements were generated with embryos from *w*^1118 ^stocks. All transposable elements were created with the pUAST plasmid and confirmed by DNA sequencing. A minimum of 50 adults for each genotype from the crosses with the *C253-gal4 *and *sca-gal4 *drivers were scored for bristle phenotypes on the thorax and head.

## Results

**Figure 1:**
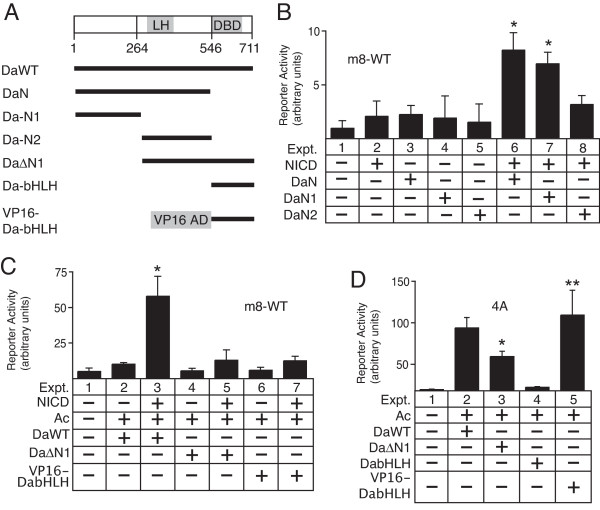
**Function of Da-N subdomains in mediating NTC-bHLH A transcriptional synergy**. **A**, diagram of Daughterless proteins used in this study. The basic-helix-loop-helix (bHLH) DNA binding domain (DBD) and "loop-helix" (LH) transcription activation domain are indicated. **B**, transcription assays with the native *m8 *promoter (*m8*-WT) and co-expression of NICD and Daughterless N-terminal protein domains. Asterisks in expts. 6 and 7 indicate promoter activity significantly greater relative to either expt. 2, 3 or 4. **C**, either deletion or replacement of the Da-N1 subdomain blocks NTC-bHLH A transcriptional synergy on the *m8*-WT promoter in S2 cells. Asterisk in expt. 3 indicates promoter activity significantly greater than observed in expt. 2. **D**, transcription activation potential of the Da-WT, Da-ΔN1 and VP16-DabHLH proteins on the artificial 4A promoter. The single asterisk in expt. 3 indicates promoter activity that is significantly greater than the basal promoter activity of the reporter plasmid (expt. 1), but less than the promoter activity observed with the co-expression of Ac/Da (expt. 2). The double asterisk in expt. 5 indicates promoter activity significantly greater than the basal promoter activity of the reporter plasmid (expt. 1), but not significantly different than the promoter activity observed with the co-expression of Ac/Da (expt. 2). For all panels, differences with p < 0.01 were considered significant.

To better understand the mechanism underlying transcriptional synergy between NTCs and bHLH A proteins, we explored the interaction between the Da bHLH A and Su(H) proteins. In the absence of the bHLH DNA-binding domain, the large N-terminal domain of Da (Da-N, Figure 1A) can synergistically activate expression of the *m8 *gene when co-expressed with the Notch intracellular domain (NICD) [1]. To determine which region within the Da-N domain is functionally important for this synergy, we divided Da-N into two subdomains (Da-N1 and Da-N2, Figure 1A) and separately co-expressed them with NICD. As shown in Figure 1B, the Da-N1 region synergistically activated the *m8 *promoter almost as well as the complete Da-N domain.

To confirm that the Da-N1 domain is critical for transcriptional synergy with NICD, we generated a Da protein lacking the Da-N1 region (Da-ΔN1, Figure 1A). The Da-ΔN1 protein was unable to synergistically activate the *m8 *promoter when co-expressed with Achaete (Ac) and NICD (Figure 1C, *cf*. expts. 3 *vs*. 5). To test whether the deletion of the Da-N1 domain eliminates the general transcription activation function of Da, rather than specifically prevent the functional interaction with NICD, we co-expressed Da-ΔN1 and Ac with the artificial 4A promoter. The 4A promoter contains 4 tandem bHLH A binding sites (A sites) adjacent to a minimal *Hsp70 *promoter. When co-expressed with Achaete, the Da-ΔN1 protein still significantly activated the 4A promoter (expt. 3, Figure 1D). These results indicated that removal of the Da-N1 region specifically prevented the functional interaction between Da and NICD that is necessary for NTC-bHLH A transcriptional synergy, but did not block the ability to activate via A sites when dimerized with Achaete.

To further test the specificity of the synergistic interaction between the Da-N domain and NICD, we generated a VP16-DabHLH fusion protein (Figure 1A). In this fusion protein, the native Da-N activation domain was replaced with the strong and constitutively active viral VP16 activation domain. Although the VP16-DabHLH protein activated the artificial 4A reporter as efficiently as Da-WT (*cf*. expts. 2 and 5 in Figure 1D), the VP16-DabHLH protein was unable to synergistically activate the *m8 *promoter when co-expressed with NICD (*cf*. expts. 2 and 3 with expts. 6 and 7 in Figure 1C). These results confirm that the Da-N domain is necessary for NTC-bHLH A transcriptional synergy, and that there is specificity to the type of transcription factor that can synergistically interact with the Su(H)/NICD binary NTC complex assembled on the SPS element [1].

**Figure 2:**
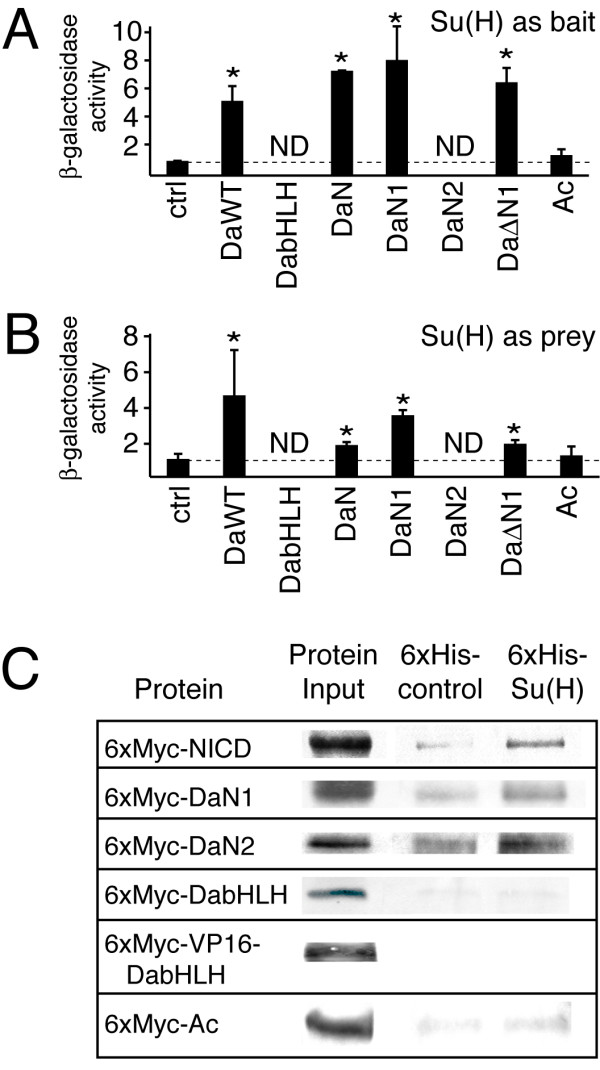
**Physical interactions of the Da-N1 and Da-N2 subdomains with Su(H)**. **A **and **B**, β-galactosidase reporter enzyme activity in yeast transformed with expression plasmids for "bait" and "prey" fusion proteins containing either Da and Su(H) proteins. Experiments with Su(H)-bait with Da-prey proteins are shown in **A**, whereas Su(H)-prey with Da-bait proteins are shown in **B**. These data indicate that the Da-N1 subdomain physically interacts with Su(H), but the interaction between DaΔN1 and Su(H) suggests that a region outside the Da-N1 domain can also interact with Su(H). Statistically significant enzyme activity (p < 0.05), indicative of a physical interaction, is denoted by an asterisk. Yeast transformants containing expression plasmids containing either the Da-bHLH or Da-N2 proteins were not viable, which is indicated by "ND" (no data). **C**, protein pull-down assays using partially purified recombinant bacterial proteins to further test whether either the Da-N1 or Da-N2 domains mediate a direct physical interaction with Su(H). These data reveal that both the Da-N1 and Da-N2 domains, but not the bHLH-C terminal domain, physically interact with CSL. NICD is a positive control which is known to interact with Su(H).

Previous yeast two-hybrid genetic studies suggested that the Da-N domain and Su(H) proteins interact as part of the NTC-bHLH A transcriptional synergy mediated by the SPS+A transcription code [1]. Additional yeast two-hybrid studies were conducted to determine whether either or both the Da-N1 and DaN2 subdomains physically interact with Su(H). Using either a "bait" or "prey" fusion Su(H) proteins, these additional studies showed that the Da-N1 interacted with Su(H) (Figure 2A and 2B). An equivalent analysis of the Da-N2 domain was prevented by the inability to transform yeast expressing either bait or prey Da-N2 fusion proteins, suggesting that the expression of the Da-N2 domain by itself was toxic to the yeast. However, the ability of the Da-ΔN1 construct to interact with Su(H) in this assay, suggests that additional regions outside the Da-N1 domain also interact with Su(H) (Figure 2A and 2B). Unfortunately, the expression of the Da-bHLH domain itself was also apparently toxic, as yeast transformants containing expression plasmids for this domain were not viable. This result indicated that both domains within the Da-ΔN1 construct (composed of the Da-N2 and Da-bHLH domains; Figure 1A) were toxic when expressed separately, which prevented determination of the domain in the Da-ΔN1 construct that interacted with Su(H).

To further test either the Da-N1 or Da-N2 domains mediate a direct physical interaction with Su(H), protein pull-down assays using partially purified recombinant bacterial proteins were used. Using 6x-His tagged Su(H) protein and 6x-Myc tagged Da proteins, both the Da-N1 and Da-N2 proteins were found to interact directly with Su(H), although the interaction with Da-N1 domain appeared to be stronger (Figure 2C). By contrast, neither the Da-bHLH domain nor the heterodimerization partner protein Ac interacted with Su(H). These results indicate that there are at least two regions in Da-N that can physically interact with Su(H).

**Figure 3:**
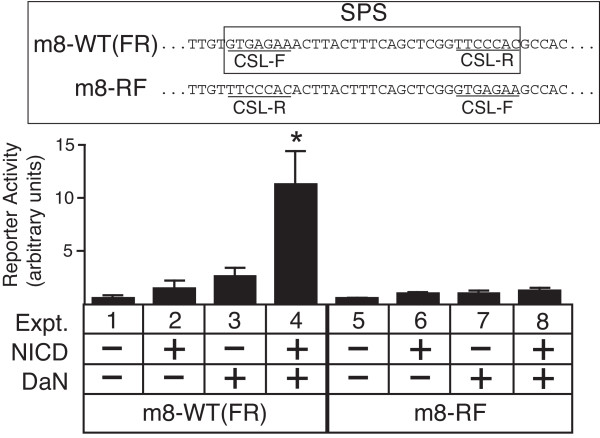
**Requirement of the SPS orientation architecture for synergistic interactions between the Da-N domain and NTCs**. Transcription assays with co-expression of NICD and Da-N protein on the wild-type *m8 *promoter (*m8*-WT, or "FR") and an *m8 *promoter containing CSL binding sites with reversed orientations relative to the wild-type promoter (*m8*-RF). Reversal of the CSL binding site orientations in the SPS element (see insert) prevents co-expressed NICD and Da-N proteins from synergistically activating the *E(spl) m8 *RF promoter, even though the Da-N domain is not bound to DNA, and can contact the Su(H) protein from any direction. Asterisk in expt. 4 indicates promoter activity significantly greater than either expt. 2 or 3 (p < 0.01).

To test whether the orientation of the NTCs protein complexes programmed by the specific inverted repeat architecture of the SPS element was critical for the interaction with the Da-N domain, we co-expressed Da-N and NICD with *m8 *promoters that contained either the wild-type Su(H) site orientations ("FR" orientations; *m8*-WT promoter) or with both Su(H) sites in orientations opposite to the wild-type (*m8*-RF promoter; see insert of Figure 3 for an explanation of Su(H) site orientations). The Da-N domain, which lacks a DNA binding domain, can synergistically activate the *m8*-WT promoter when co-expressed with NICD (Figure 3). Since the Da-N protein is not tethered to the DNA and can contact the Su(H) protein from any direction, the ability of the Da-N protein to synergize with the Su(H)/NICD complex need not be constrained by orientation of the Su(H) sites. Strikingly, however, the Da-N protein cannot synergize with NICD to activate *m8*-RF promoter (*cf*. expts 4 *vs*. 8 in Figure 3). These results suggest that the architecture of the wild-type Su(H) binding site orientations in the SPS element results in the formation or exposure of an interaction domain in Su(H) (as part of the NTC protein complex) that is critical for the physical interaction with Da-N domain. Although our previous analysis showed that Su(H) protein binds equally well to both the *m8*-WT and and *m8*-RF S site architectures [1], we cannot exclude the possibility that other mechanisms, such as a diminished stability of Su(H)/NICD complex or an impaired recruitment of co-activators such as Mastermind, also contribute to the loss of *Notch*-bHLH A transcriptional synergy on the *m8*-RF promoter.

To test the *in vivo *function of the Da-N domain, we over-expressed wild-type and mutant forms of Da in transgenic flies using the Gal4-UAS driver system [5]. Previous studies have shown that ubiquitous over-expression of Da is embryonic lethal [6]. Therefore, we limited the over-expression of Da protein to proneural clusters using the *C253-gal4 *[7] and *sca-gal4 *driver lines [8].

**Figure 4:**
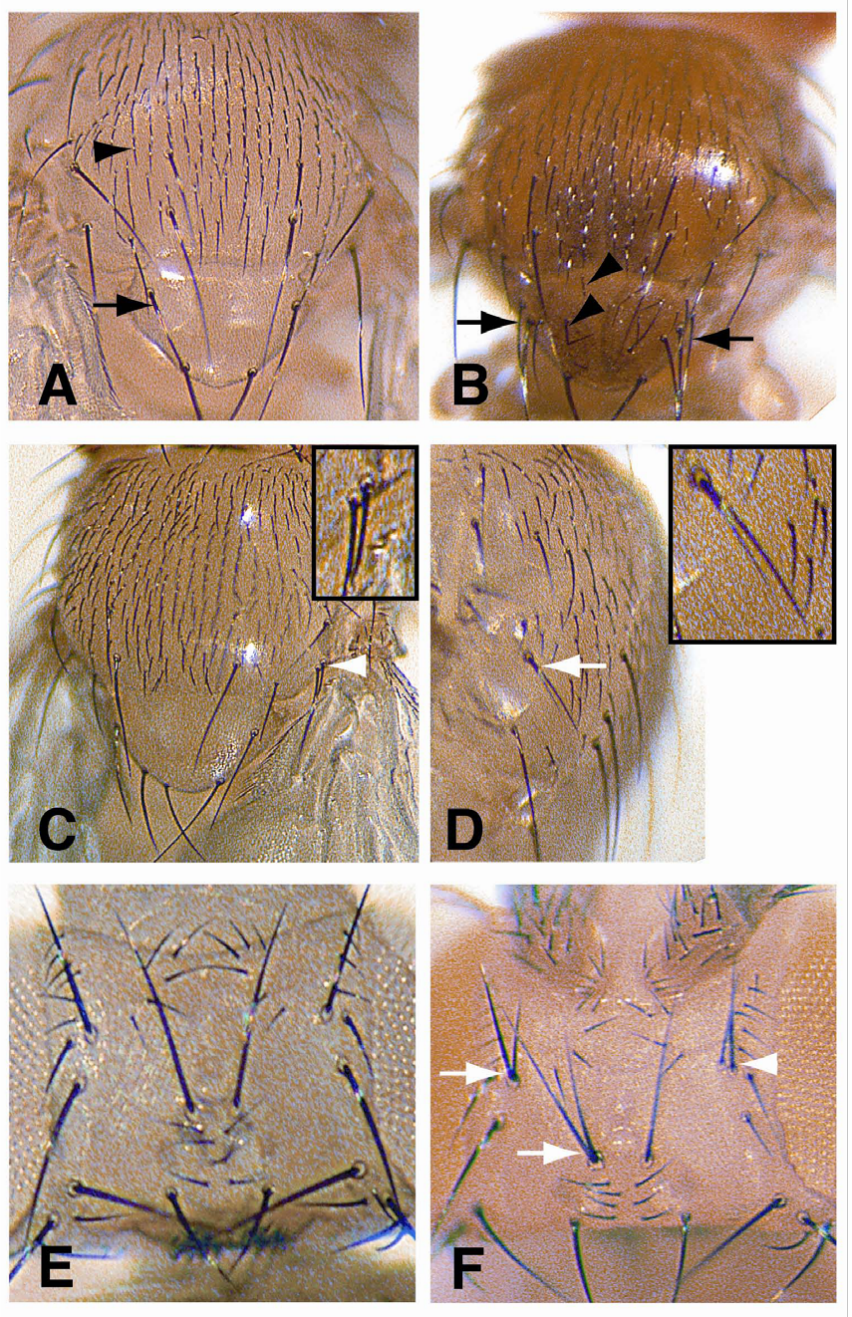
**Bristle analysis of transgenic flies over-expressing various Da proteins in proneural clusters *in vivo***. **A**, pattern of large (macrochaete; black arrow) and small (micochaete; black arrowhead) bristles on the wild-type thorax. **B**, thorax of a fly with the *C253-gal4 *driver over-expressing Da-WT contains numerous ectopic macrochaete (black arrows) and microchaetae on the scutellum (black arrowheads; compare with **A**). **C **and **D**, the thorax of flies over-expressing Da-bHLH with the *C253-gal4 *driver contain extra macrochaete, including some that are immediately adjacent to other macrochaete (white arrowhead and insert in **C**), and bristles with twinned shaft/bristle cells (white arrow and insert in **D**). **E**, bristle pattern of the wild-type head. **F**, the head of a fly over-expressing Da-bHLH with the *sca-gal4 *driver contain extra bristles that are immediately adjacent to other macrochaete (white arrowhead) and bristles with twinned shaft/bristle cells (white arrows). The presence of extra macrochaetes immediately adjacent to normal macrochaetes is characteristic of defects in *Notch *signalling.

Although Da is necessary for both bHLH A and bHLH R gene expression in proneural clusters, over-expression of Da-WT *in vivo *generated bristle phenotypes consistent with an increase in proneural bHLH A function. With both driver lines, several additional macrochaete and microchaete were observed on the thorax of all the flies examined (*cf*. Figure 4A*vs*. 4B). By contrast, missing bristles were not observed, which would be expected if the over-expression of Da-WT generated persistent high expression levels of bHLH R genes [9]. Importantly, in almost all cases, nearly all bristles were visibly separated from each other and rarely found to be immediately adjacent to each other, suggesting that Notch mediated lateral inhibition is still normally functioning.

These observations suggest that the over-expression of Da-WT increased proneural activity without disrupting Notch-mediated lateral inhibition. Previous studies in embryos have indicated that Da is rate-limiting, such that over-expression of Achaete-Scute proteins does not give rise to overproduction of NPCs unless Da is also over-expressed [6]. Thus, the over-expression of Da-WT is likely to sensitize the epithelium, resulting in the formation of ectopic PNCs. Alternatively, or in addition, the observed ectopic mechanosensory bristles may result from an expansion of the proneural territories capable of generating NPCs [10]. Another alternative mechanism, is that Da can also physically interact with a subset of *E(spl)-C *bHLH R proteins which may inhibit their repression function [11,12].

Both the transcription and protein interaction assays in this study indicated that the Da-N domain was critical for synergistic activation of *m8*. To test whether the Da-N domain was critical for lateral inhibition *in vivo*, we over-expressed the Da-bHLH protein. Because both the Da-N1 and Da-N2 subdomains physically interacted with Su(H), the Da mutant lacking the entire Da-N domain (the Da-bHLH protein) was studied. With both driver lines, almost all flies (~90%) contained extra bristles and/or bristles with twin shaft cells on the thorax (Figure 4C and 4D). Most flies (~80%) with *sca-gal4 *driver also had both extra bristles and bristles with twinned shafts on the head (Figure 4F). However, unlike over-expression with Da-WT, the extra macrochaete on the thorax and head observed with Da-bHLH over-expression were not in ectopic locations. Rather, these extra bristles were all near other macrochaetae, and, in many cases, the extra bristles were immediately adjacent to other bristles (Figure 4C and 4F). The lack of ectopic bristles outside of proneural regions indicates that the over-expression of Da-bHLH did not expand the proneural territories or sensitize the epithelium as did Da-WT. Rather, the over-expression of Da-bHLH specifically disrupted lateral inhibition within the normal proneural clusters apparently by acting as a dominant negative protein. This disruption allowed adjacent bristles to form, which are normally prevented by Notch-mediated lateral inhibition. Together, these findings are consistent with the specific role of the Da-N domain that we have identified for selective activation of *E(spl)-C *bHLH R gene expression during *Notch *signalling in proneural clusters *via *the SPS+A transcription code.

The over-expression either the Da-N, Da-N1or Da-N2 proteins in transgenic flies, as reciprocal experiments to the over-expression of the Da-bHLH protein, were predicted to generate a bristle-loss phenotype. However, transgenic over-expression of either these proteins using the same drivers used for the Da-bHLH experiment did not generate any abnormal bristle phenotypes (data not shown). These observations do not necessarily contradict the conclusion that the DaN domain is critical for selective activation of *E(spl)-C *bHLH R gene expression during *Notch *signalling in proneural clusters. Rather, the transcription assay data with the Da-N, Da-N1 and Da-N2 proteins suggest that these proteins are too weak as transcription activators to produce phenotypes that can be observed in vivo. For example, by comparing expt. 6 in Figure 1B*vs*. expt. 3 in Figure 1C, one can see that the DaN protein mediated reporter activity that was over 5 times less than the DaWT protein. The DNA-bound complexes formed with Su(H) and either Da-N, Da-N1 or Da-N2 proteins may substantially weaker relative to complexes with Su(H) and DaWT since the Da-N, Da-N1 and Da-N2 proteins lack a functional DNA binding domain. An alternative, but not mutually exclusive explanation, is that the Da-N, Da-N1or Da-N2 proteins themselves are less stable or have a higher turn-over than either the full length DaWT or DabHLH proteins in transgenic flies. For either explanation, the transcriptional activation mediated by the Da-N, Da-N1 and Da-N2 proteins was observed in the cultured cell transcription assays presumably because of the high sensitivity of the luciferase reporter assay system.

## Summary and conclusion

The current study indicates that direct physical interactions between the Su(H) and Da proteins are critical for the co-activation of Su(H)/NICD complexes by bHLH A proteins on SPS+A modules. Although our previous yeast two-hybrid genetic-based studies reported evidence for a potentially direct interaction between these proteins [1], the current study both identified two distinct N-terminal subdomains of Da each of which interact with Su(H), and showed that deletion of these regions is sufficient to eliminate synergistic activation of *m8 *in transcription assays. Moreover, the transcription assays with the Da-N construct (containing both Su(H) interaction domains, but no bHLH domain) suggest that the native SPS orientation-architecture functions to induce the formation of, or expose, an interaction surface on Su(H) with which the Da-N domain interacts. Reversal of the Su(H) binding site orientations in the SPS may disrupt the Su(H)-Da interaction by either creating steric hindrance that obstructs Da-N from binding Su(H), or by changing the overall Su(H) protein conformation such that the relevant interaction surface no longer forms and Da-N cannot bind Su(H). The over-expression of a Da construct lacking both Su(H)-interacting regions resulted in disruption of Notch-mediated lateral inhibition during mechanosensory bristle neurogenesis, Together, these experiments confirm the functional importance of the Da-N domain in mediating Notch-proneural transcriptional synergy during neurogenesis in vivo.

## Competing interests

The authors declare that they have no competing interests.

## Authors' contributions

LX and JWC performed all the experiments. MAC and JWC designed, analyzed and interpreted of all experiments, as well as wrote the manuscript. All authors read and approved the final manuscript.
